# Infection diagnosis in hydrocephalus CT images: a domain enriched attention learning approach

**DOI:** 10.1088/1741-2552/acd9ee

**Published:** 2023-06-16

**Authors:** Mingzhao Yu, Mallory R Peterson, Venkateswararao Cherukuri, Christine Hehnly, Edith Mbabazi-Kabachelor, Ronnie Mulondo, Brian Nsubuga Kaaya, James R Broach, Steven J Schiff, Vishal Monga

**Affiliations:** 1 Department of Electrical Engineering, the Pennsylvania State University, University Park, PA 16801, United States of America; 2 Center for Neural Engineering, the Pennsylvania State University, University Park, PA 16801, United States of America; 3 College of Medicine, the Pennsylvania State University, University Park, PA 16801, United States of America; 4 CURE Children’s Hospital of Uganda, Mbale, Uganda; 5 Department of Neurosurgery, Yale University, New Haven, CT 06510, United States of America

**Keywords:** machine learning, neural network, attention learning, hydrocephalus, CT, treatment planning, skull stripping

## Abstract

**Objective.:**

Hydrocephalus is the leading indication for pediatric neurosurgical care worldwide. Identification of postinfectious hydrocephalus (PIH) verses non-postinfectious hydrocephalus, as well as the pathogen involved in PIH is crucial for developing an appropriate treatment plan. Accurate identification requires clinical diagnosis by neuroscientists and microbiological analysis, which are time-consuming and expensive. In this study, we develop a domain enriched AI method for computerized tomography (CT)-based infection diagnosis in hydrocephalic imagery. State-of-the-art (SOTA) convolutional neural network (CNN) approaches form an attractive neural engineering solution for addressing this problem as pathogen-specific features need discovery. Yet black-box deep networks often need unrealistic abundant training data and are not easily interpreted.

**Approach.:**

In this paper, a novel brain attention regularizer is proposed, which encourages the CNN to put more focus inside brain regions in its feature extraction and decision making. Our approach is then extended to a hybrid 2D/3D network that mines inter-slice information. A new strategy of regularization is also designed for enabling collaboration between 2D and 3D branches.

**Main results.:**

Our proposed method achieves SOTA results on a CURE Children’s Hospital of Uganda dataset with an accuracy of 95.8% in hydrocephalus classification and 84% in pathogen classification. Statistical analysis is performed to demonstrate that our proposed methods obtain significant improvements over the existing SOTA alternatives.

**Significance.:**

Such attention regularized learning has particularly pronounced benefits in regimes where training data may be limited, thereby enhancing generalizability. To the best of our knowledge, our findings are unique among early efforts in interpretable AI-based models for classification of hydrocephalus and underlying pathogen using CT scans.

## 1. Introduction

### 1.1. Introduction to the problem

Hydrocephalus is the leading indication for pediatric neurosurgical care worldwide, with the majority of the pediatric hydrocephalus burden falling on the developing world [1]. Over half of the pediatric hydrocephalus cases in regions such as sub-Saharan Africa appear to be postinfectious hydrocephalus (PIH) in nature [2], while non-postinfectious hydrocephalus (NPIH) cases have etiologies such as hemorrhage or congenital malformations [1]. Etiologies such as congenital malformations in particular lead to distinct anatomic pathology in brain imaging. Infectious pathology can be diverse in hydrocephalic patients, and is characterized by calcifications, abscesses, loculations and debris in the cerebral ventricles (cerebrospinal fluid filled cavities within the brain) [3]. Distinguishing PIH from NPIH is critical since a child with an active infection within the brain may not have clear signs of infection such as fever, yet performing surgery to divert the excess fluid of hydrocephalus should wait until the infection has properly treated with antimicrobials. Despite the different underlying pathophysiology behind these types of hydrocephalus, the major imaging pathology seen is dilation of the cerebral ventricles, and there are many other shared features such as edema within the brain tissue and enlarged overall head size.

**Figure 1.**
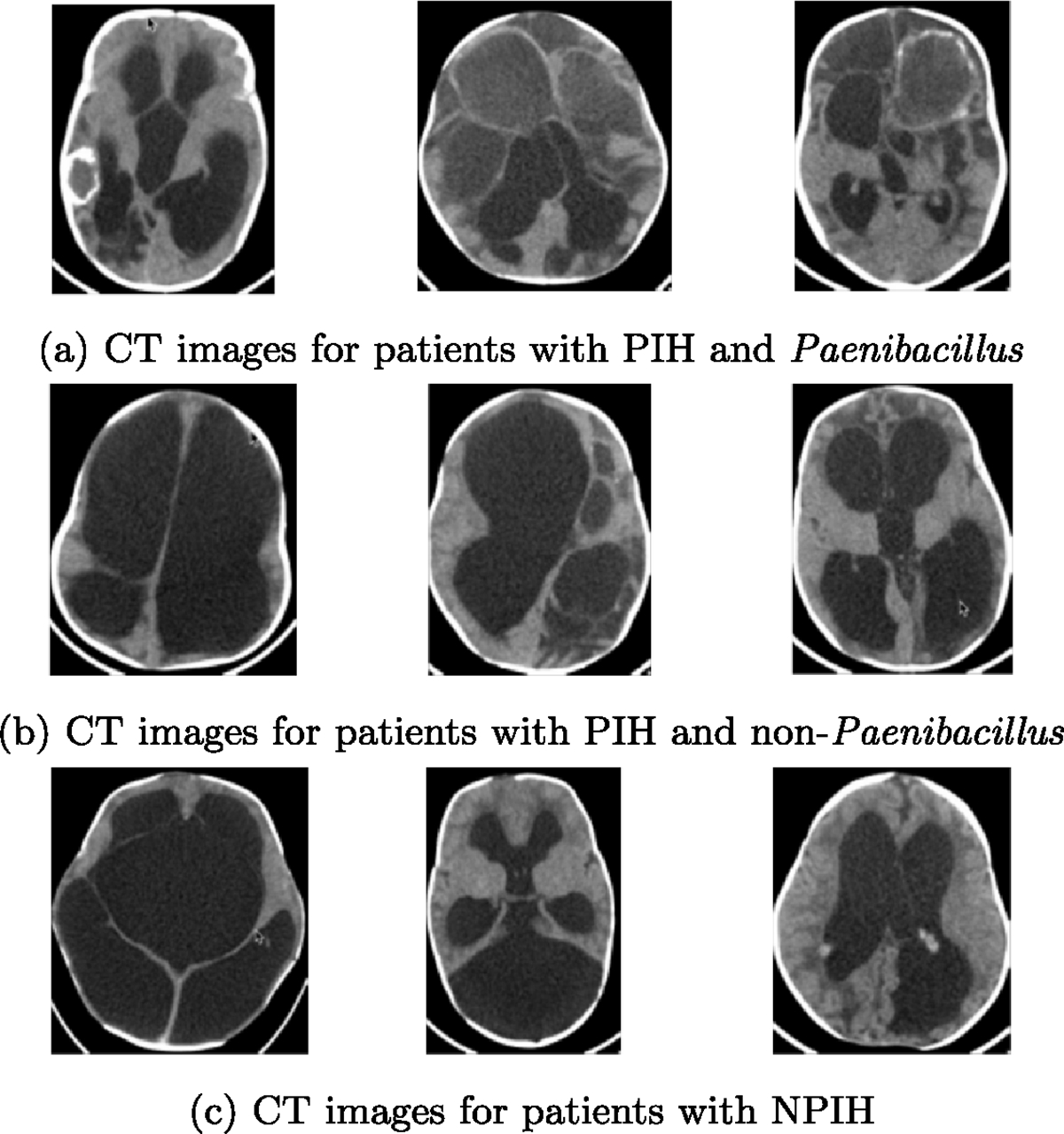
CT images from the CURE dataset.

In high resource medical systems, magnetic resonance imaging (MRI) is used to diagnose the etiology of hydrocephalus and plan surgical treatment. However, in the developing world brain computerized tomography (CT) scans when available are the main imaging technology used for these purposes. The expertise of a medical professional is necessary to diagnose the underlying hydrocephalus etiology, so that the treatment plan can be developed appropriately. Even in a setting with radiologists who can attempt to differentiate PIH/NPIH, the infectious pathogen underlying PIH can be difficult to determine. There is a newly described disease in neonates—Neonatal Paenibacilliosis—that is highly lethal and is the most common pathogen that leads to postinfectious hydrocephalus in survivors [4]. Recent publications [3] found that *Paenibacillus* bacteria most commonly leads to severe PIH in Uganda making *Paenibacillus* infection to be correlated with more severe imaging pathology in terms of abscesses, calcifications, and other infectious processes [3]. A few examples of CT images from NPIH patients and PIH patients with and without *Paenibacillus* are shown in figure 1. Diagnosing *Paenibacillus* is important for its special antibiotic treatment requirements.

**Figure 2.**
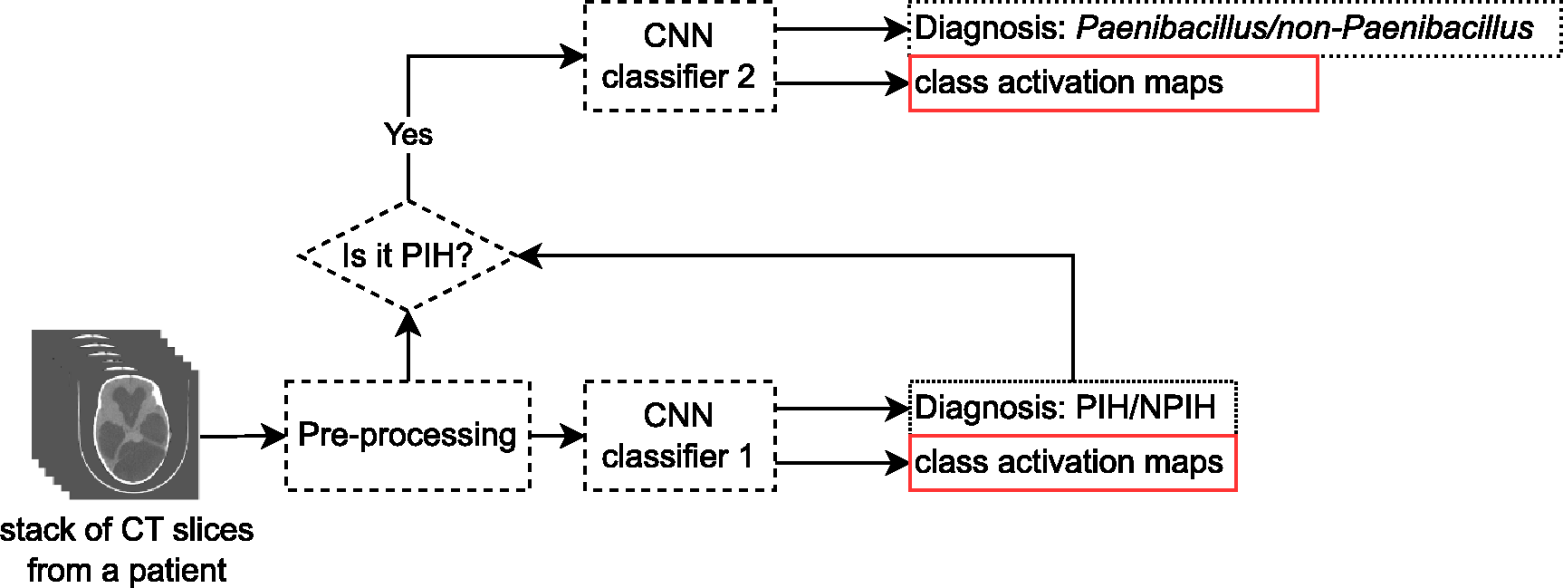
Pipeline of infection diagnosis for the hydrocephalus and pathogen diagnosis problem.

Hence, this work aims to develop an automatic brain CT diagnosis system for PIH/NPIH with a *Paenibacillus* detection model that would support the diagnostics of medical experts and laboratories and improve the management of pediatric hydrocephalus. The flow chart in figure 2 illustrates the objective of our work: given CT images of a patient, we would like our convolutional neural network (CNN) classifier 1 to classify whether a patient has PIH or NPIH. Class activation maps (CAMs) are also desired for clinicians to know which regions contribute to the model’s decision and which regions do not [5]. If the outcome of the classification is PIH, the same images are sent to classifier 2 for pathogen classification i.e. *Paenibacillus*/non-*Paenibacillus*, with CAMs output for this decision as well.

Development of such a CT image classifier is challenging. In traditional computer-aided diagnosis systems (CADs), manually extracting features from the images is always required for subsequent classifiers such as support-vector machine. In [6], the authors developed a thresholding technique to extract features from brain MRI images and applied a SVM to classify cerebral microbleeds. This technique is only compatible with very well studied pathogens or features. In recent years, deep learning-based methods have shown remarkable performance in medical image classification or detection [7–15].

The existing works can be approximately categorized as (1) 2D approaches [9, 16–20], which take as input 2D slices; (2) 3D approaches [7, 8, 11, 21, 22], which take as input 3D volume; (3) multi-plane approaches [10, 12, 23, 24], which fuse 2D slices from multiple planes; (4) hybrid of 2D/3D approaches [25–27], which combine 2D and 3D approaches. Although these methods exhibit good performance in specific aspects, they still suffer from limitations: in 2D and multi-plane approaches, 3D context information is not fully explored; for 3D approaches and hybrid methods, training such complex model architectures is challenging. Furthermore, the hydrocephalus brain CT scans have the following characteristics that make the classification more challenging:

1. The condition of hydrocephalus varies greatly between different patients in terms of pathology.
2. Different etiologies of infection often have many features in common, which hinders the ability of a CNN to find strong discriminative features between classes.

Due to these characteristics, when there is limited training data available, the deep models may over-fit the training data which means the model ‘memorizes’ the training samples. Therefore, it learns to activate non-diagnostic regions in the image as long as they are similar to seen training data rather than the actual pathological patterns.

To address the challenges, we propose an AI image classifier regularized by a novel **brain attention regularizer** (BAR), which will suppress the model’s attention outside the brain region. Furthermore, a 2D/3D hybrid model with a collaborative training strategy is proposed to mine and to combine discriminative features in higher dimensions, in which the relation of 2D slice and 3D volume is explored and 2D and 3D branches collaborate with each other.

## 2. Material and methods

### 2.1. Dataset

The dataset was collected at a nationwide neurosurgical referral hospital, CURE Children’s Hospital of Uganda (CCHU), in Mbale in the eastern region of the country. Infants were recruited as described in [3, 28]. Infants were eligible for inclusion in the study if they were under 3 months old, diagnosed with hydrocephalus, and their mothers were at least 18 years of age and able to provide informed consent in a language they understood.

Inclusion criteria for PIH: (a) weight >2500 g, (c) no history consistent with hydrocephalus at birth, and either (i) a history of febrile illness and/or seizures preceding the onset of clinically apparent hydrocephalus, or (ii) alternative findings such as imaging and endoscopic results indicative of prior ventriculitis including septations, loculations, or deposits of debris within the ventricular system.

Inclusion criteria for NPIH: (a) weight >2500 g, (b) findings of non-infectious origin of hydrocephalus on CT scan or at endoscopy such as a lesion obstructing the Aqueduct of Sylvius such as tumor or cyst, aneurysm, or cavernous malformation, Dandy-Walker cyst, or other congenital malformation of the nervous system, or (d) evidence of hemorrhage as cause of hydrocephalus such as (i) bloody cerebrospinal fluid and (ii) absence of findings consistent with PIH or congenital origin of hydrocephalus. Exclusion criteria: (a) prior surgery on the nervous system (shunt, third ventriculostomy, or myelomeningocele closure), or (2) evidence of communication of nervous system with skin such as meningocele, encephalocele, dermal sinus tract, fistula, etc.

We collected computed tomography (CT) images from 387 infants labeled as PIH (206/387) and NPIH (181/387) based on the criterion introduced above. Each scan set contains around 30–50 slices. Within the PIH cohort, 90 scan sets are labeled as *Paenibacillus* and 106 scan sets are labeled as non-*Paenibacillus* by quantitative polymerase chain reaction [3].

### 2.2. Pre-processing

#### 2.2.1. skull stripping

**Figure 3.**
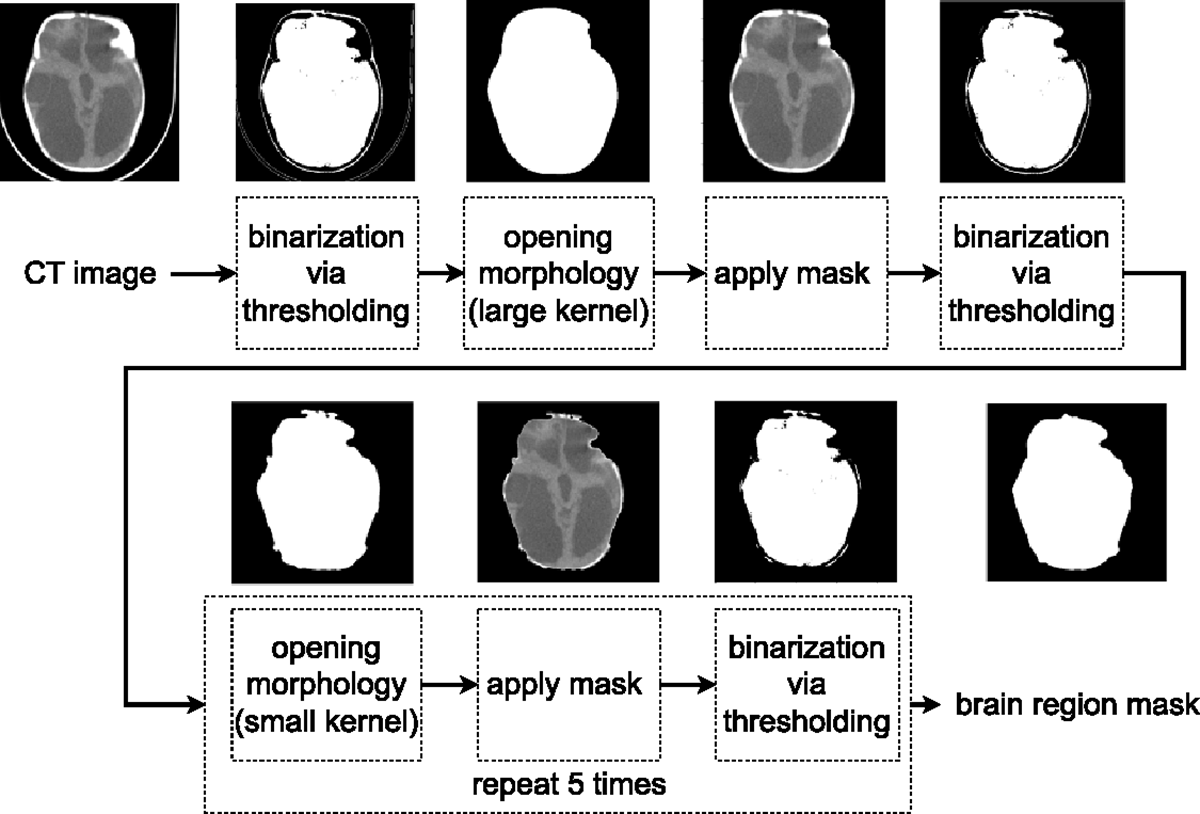
The diagram illustrates the steps involved in skull stripping CT images. Initially, a binary mask is created by applying a threshold to the CT image, followed by an opening morphology using a large kernel. Subsequently, the brain region mask is obtained by performing five iterations of thresholding and opening morphology using small kernels.

We generate brain region masks using a fully automated and unsupervised skull stripping algorithm, developed in a similar manner to [29]. It takes 0.016 seconds to skull strip a CT image with dimensions of 448 × 448. The procedures of skull stripping are shown in figure 3. We verified experimentally that the extracted skull region has near perfect overlap with expert annotated skull masks.

#### 2.2.2. Inter-slice thickness adjustment

To account for potential variations in inter-slice thickness between scans, we adjusted all scans in our dataset to have a standardized inter-slice thickness of 3.6 mm, which was the average thickness in our dataset. To achieve this, we used the thickness (t) between slices from the DICOM [30] metadata of each CT stack. The original cube, which was sized at M×448×448, was then resized to $\left\lfloor M\cdot \frac{t}{3.6}\right\rfloor \times 448\times 448$. This adjustment ensured that all CT stacks had a consistent inter-slice thickness. The steps are as below:

- Extract the sagittal and coronal slices of dimensions M×448 from the original cube and interpolate them to a new size of $\left\lfloor M\cdot \frac{t}{3.6}\right\rfloor \times 448$ using k-nearest neighbor interpolation.
- Create cubes with dimensions $\left\lfloor M\cdot \frac{t}{3.6}\right\rfloor \times 448\times 448$ from the interpolated sagittal and coronal slices obtained in step (i), and calculate the average of these two cubes. This allows us to combine the information from both directions; finally we extract and process axial slices from this re-sized data cube.

### 2.3. Brain attention regularized network

#### 2.3.1. Motivation

Interpretability of decisions made by deep learning models is crucial and desired by clinicians for treatment planning. In [31], a CAM for image classification is computed by taking a weighted average of the feature maps given by:

(1) $$A^{k}=\sum_{i=1}^{C}F_{i}\cdot w_{i}^{k}$$

where k represents the *k*^th^ class, $F_{i}$ is the *i*^th^ feature map from the last convolution layer, C is the number of feature maps and $w_{i}^{k}$ is the parameter of the fully connected layer that weighs the importance of the *i*^th^ feature map in class k. The highly weighted regions in $A^{k}$ contribute more towards prediction of the class k.

**Figure 4.**
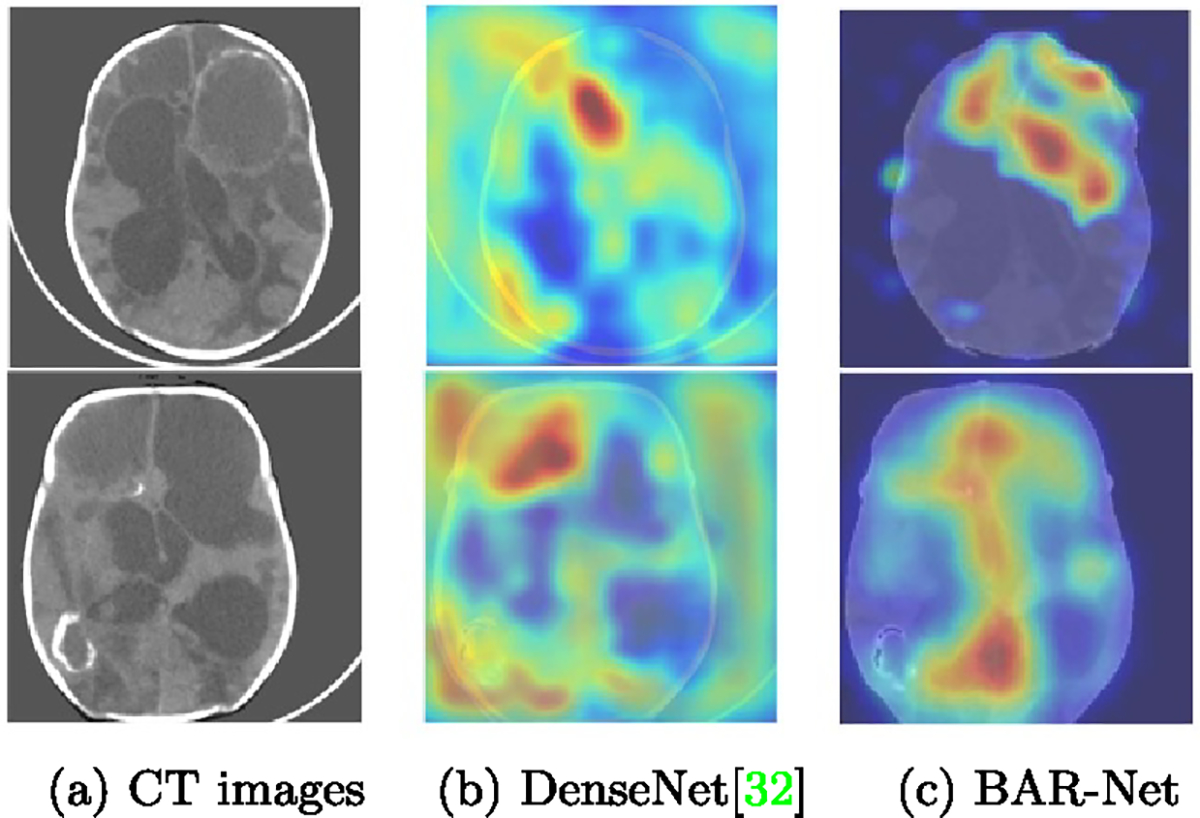
Inputs and CAMs of deep networks (upscaled for visualization). The activations outside brain regions in (b) are not interpretable.

Figure 4(b) demonstrates that when standard deep CNN models, such as DenseNet [32], are used, there are often significant activations outside the region of interest (i.e. the brain in our case). These spurious activations outside the brain are uninterpretable and may raise concerns for clinicians regarding the reliability of the decision-making process of deep networks. To address this issue, we propose a BAR that suppresses the activations outside the brain, resulting in the activations shown in figure 4(c).

#### 2.3.2. Structure of BAR-Net

**Figure 5.**
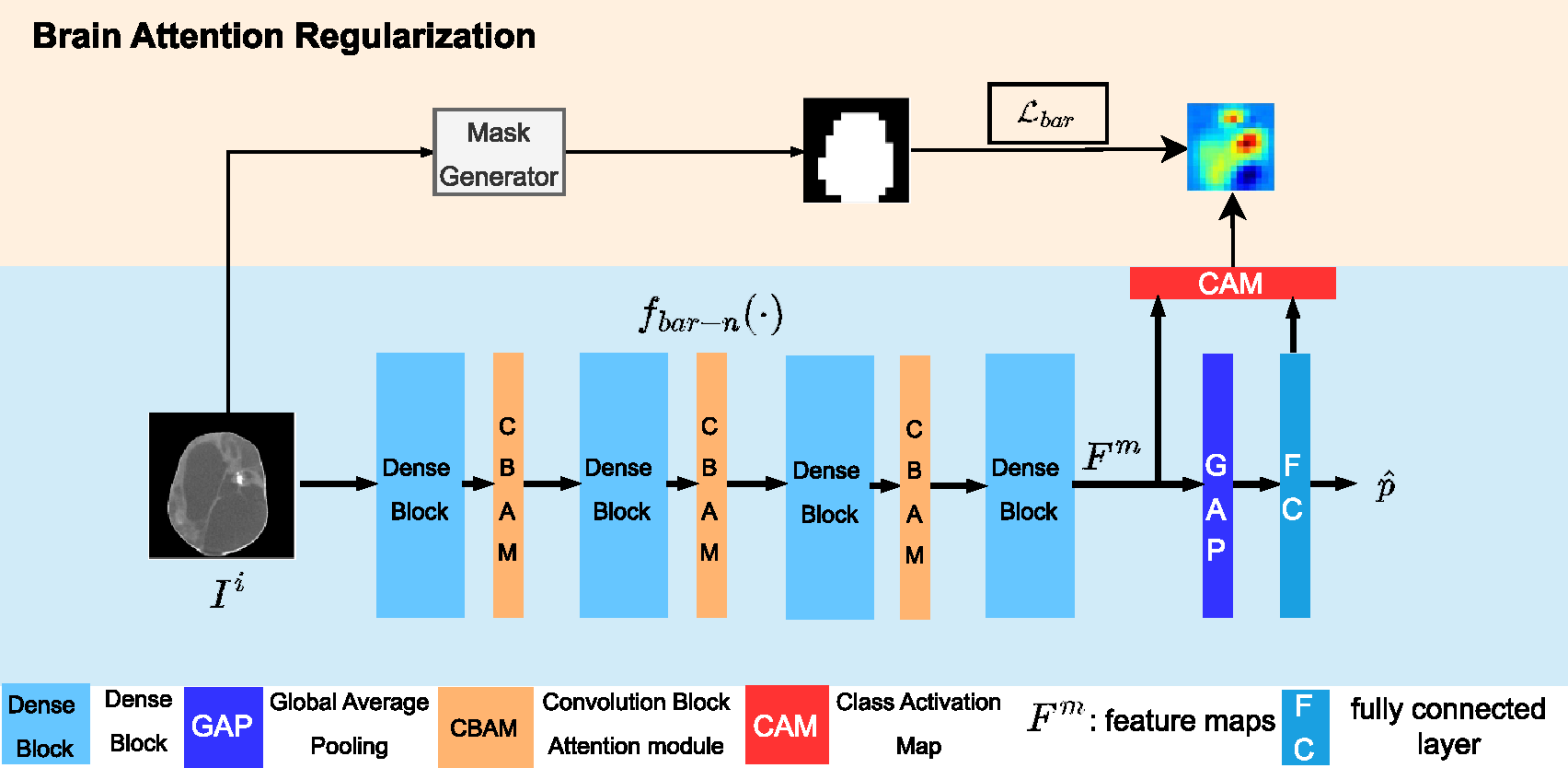
Architecture of brain attention regularized network, which consists of a main classification network and a branch for brain attention regularization. CAM is output and regularized using the brain region masks.

Our proposed brain attention regularized network is illustrated in figure 5. The backbone of the BAR-Net is a DenseNet [32], which is composed of a 2D convolution layer, 4 dense blocks, 3 convolution block attention modules [33], and 3 transition blocks. Each dense block comprises several densely connected convolutional layers, a rectified linear unit (ReLU) activation function, and a batch normalization layer. Finally, feature vectors are fed to a fully connected layer for classification.

The loss function used for training the BAR-Net is the standard binary cross-entropy (BCE) loss ${ℒ}_{BCE}$, which is given by:

(2) $${ℒ}_{BCE}(y,\overset{ˆ}{p})=-ylog(\overset{ˆ}{p})-(1-y)log(1-\overset{ˆ}{p})$$

where y is the binary label, and $\overset{ˆ}{p}$ is BAR-Net’s output probability prediction.

#### 2.3.3. Brain attention regularization

To restrict the model’s attention towards the more discriminative patterns that are naturally present inside the brain region, a new loss term ${ℒ}_{\text{bar}}$ is introduced with the help of the brain region masks M which are downsized to the same size as CAMs. The brain region masks are defined as:

(3) $$M(x,\;y)=\left\{\begin{array}{ll} 1, & \text{if}\;(x,y)\;\text{is}\;\text{inside}\;\text{brain}\;\text{region}\\ 0, & \text{if}\;(x,y)\;\text{is}\;\text{outside}\;\text{brain}\;\text{region} \end{array}.\right.$$

The BAR ${ℒ}_{\text{bar}}$ is given by:

(4) $${ℒ}_{\text{bar}}=∥\left(1-M\right)⊙A{∥}_{1},$$

where A is the CAM and ⊙ represents the (elementwise) Hadamard product. The activation outside the brain is collected and will be suppressed by minimizing this term. Intuitively this loss term enforces the model to pay attention inside the brain region.

The combined loss function for the BAR-Net is hence given by

(5) $${ℒ}_{\text{bar}-\text{n}}={ℒ}_{\text{BCE}}+\alpha {ℒ}_{\text{bar}},$$

where α is a hyper-parameter balancing the weight of the regularization term. Training the model using this loss function will improve the model’s classification performance with the model’s attention regularized within the brain.

As observed from figure 4(c), BAR-Net infers the final decision based on regions inside brain, resulting in more informative patterns and thereby improving the robustness of the network.

#### 2.3.4. Ensemble of predictions

It is important to note that each CT scan set contains multiple slices that span from the base to the top of the head. However, typically, the slices in the middle region (which is approximately $\frac{1}{3}$ of the total number of slices) are the most informative for infection diagnosis. Therefore, we use an ensemble of probability scores:

(6) $$\hat{p}=\frac{1}{N_{2}-N_{1}}\sum_{i=N_{1}}^{N_{2}}f_{\text{bar-n}\;}\left(I^{i}\right)$$

where $I^{i}$ is the *i*^th^ slice, $N_{1}=\lfloor N/3\rfloor ,$ $N_{2}=\lfloor 2\cdot N/3\rfloor$, and $f_{\text{bar}-n}(\cdot )$ is the functional representation of BAR-Net as shown in figure 5.

Equation (6) is equivalent to assigning less weights to the slices that the model is less certain about (i.e. the information is more ambiguous) – this is known to induce less bias than majority voting [34].

### 2.4. Collaborative brain attention regularized network (CBAR-Net)

#### 2.4.1. Motivation

The 2D BAR-Net can leverage pre-trained models and regularized learning as mentioned in section 2.3. However, a shortcoming of the 2D ensemble strategy is the incapability of exploiting inter-slice information. The alternative is to learn 3D networks [7, 8, 35, 36] that are becoming popular in medical imaging involving 3D data, but invariably require large amounts of training data. In practice, the problem is exacerbated by the fact that pre-trained models for 3D deep networks are much less available than those for their 2D counterparts. Hence, a mechanism that is able to combine the advantages of both methods and mitigate their respective disadvantages is desirable.

Motivated by the complementary properties of 2D and 3D models, a hybrid of 2D and 3D BAR-Nets is proposed to address these issues, in which a 2D branch and a 3D branch are trained jointly in a collaborative way. Hence, the proposed model is called CBAR-Net.

#### 2.4.2. Structure of CBAR-Net

**Figure 6.**
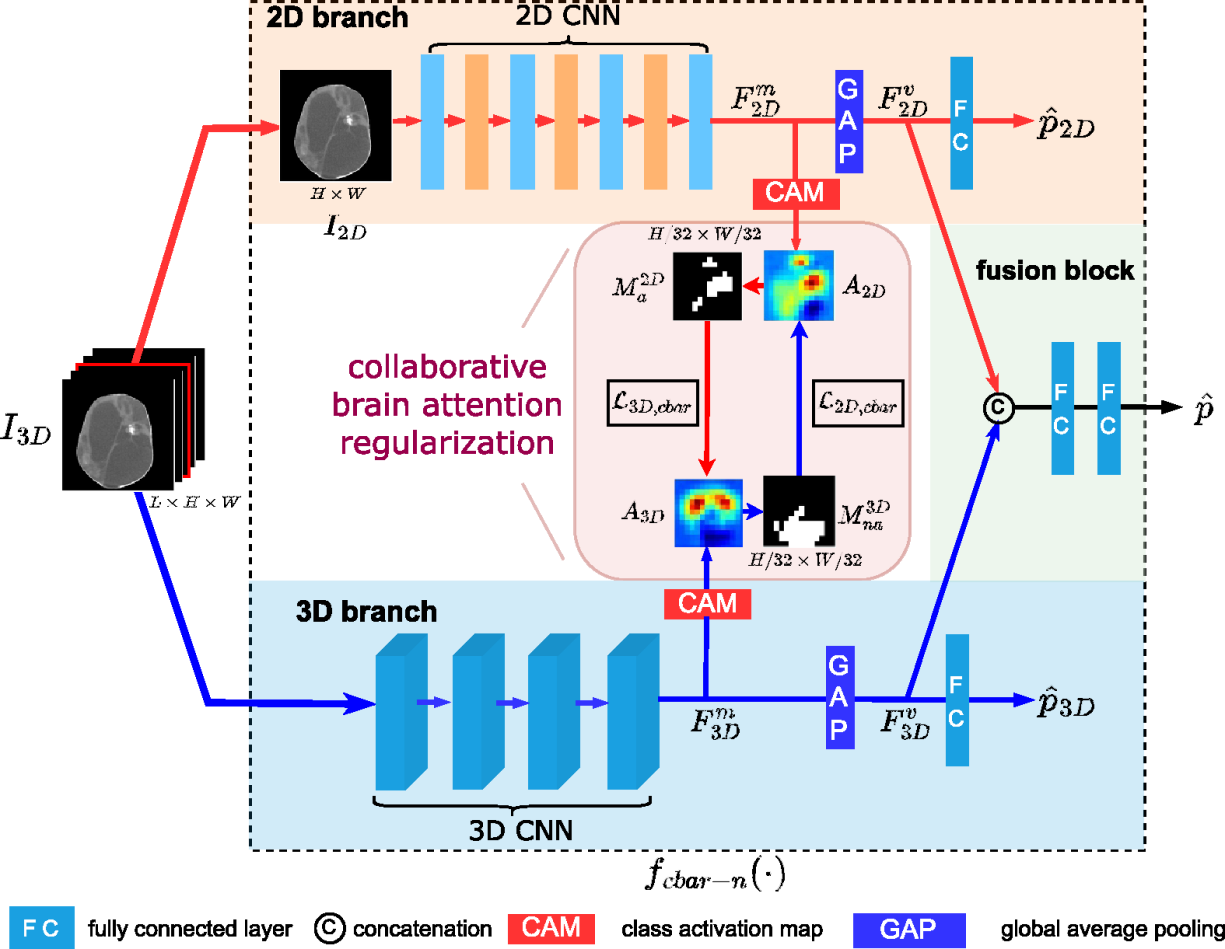
Architecture of collaborative brain attention regularized network, which consists of a 2D branch, a 3D branch and a fusion block. The input to the CBAR-Net is a CT stack (sub-volume) where the middle slice is fed to 2D branch and the whole stack is fed to 3D branch. The features extracted from two branches are fused for final classification. Meanwhile, two branches collaborate with each other via the novel collaborative brain attention regularization.

The architecture of CBAR-Net is illustrated in figure 6, which consists of a 2D branch, a 3D branch and a fusion block.

- *3D branch* The 3D branch is a 3D version of the BAR-Net, where the input is a 3D cube $I_{3D}$ with dimensions of L×H×W. It produces probability scores ${\overset{ˆ}{p}}_{3D}$, feature vectors $F_{3D}^{v}$ and maps $F_{3D}^{m}$, and CAM $A_{3D}$ with dimensions of $\frac{L}{16}\times \frac{H}{32}\times \frac{W}{32}$. In our case, L is set to 16 for two reasons: Firstly, using too few slices would not capture enough inter-slice information, while using more slices would increase the dimension of the inputs without adding more useful information. 16 slices are sufficient to cover most of the informative region. Secondly, setting L=16 leads to the same dimensions for $A_{2D}$ and $A_{3D}$, which is beneficial for our regularization described later. Further ablation studies are presented in section 3.2.
- *2D branch* The 2D branch has a similar architecture to the BAR-Net, where the input is the center slice $I_{2D}$ with dimensions of H×W extracted from the 3D input $I_{3D}$. It produces probability scores ${\overset{ˆ}{p}}_{2D}$, feature vectors $F_{2D}^{v}$ and maps $F_{2D}^{m}$, and CAM $A_{2D}$ with dimensions of $\frac{H}{32}\times \frac{W}{32}$.
- *fusion block* The fusion block concatenates the feature vectors $F_{2D}^{v},$ $F_{3D}^{v}$ outputted from 2D and 3D branches and maps it to probability scores $\overset{ˆ}{p}$ through fully connected layers and an ReLU activation function.

#### 2.4.3. Relation of 2D/3D branch’s activation

**Figure 7.**
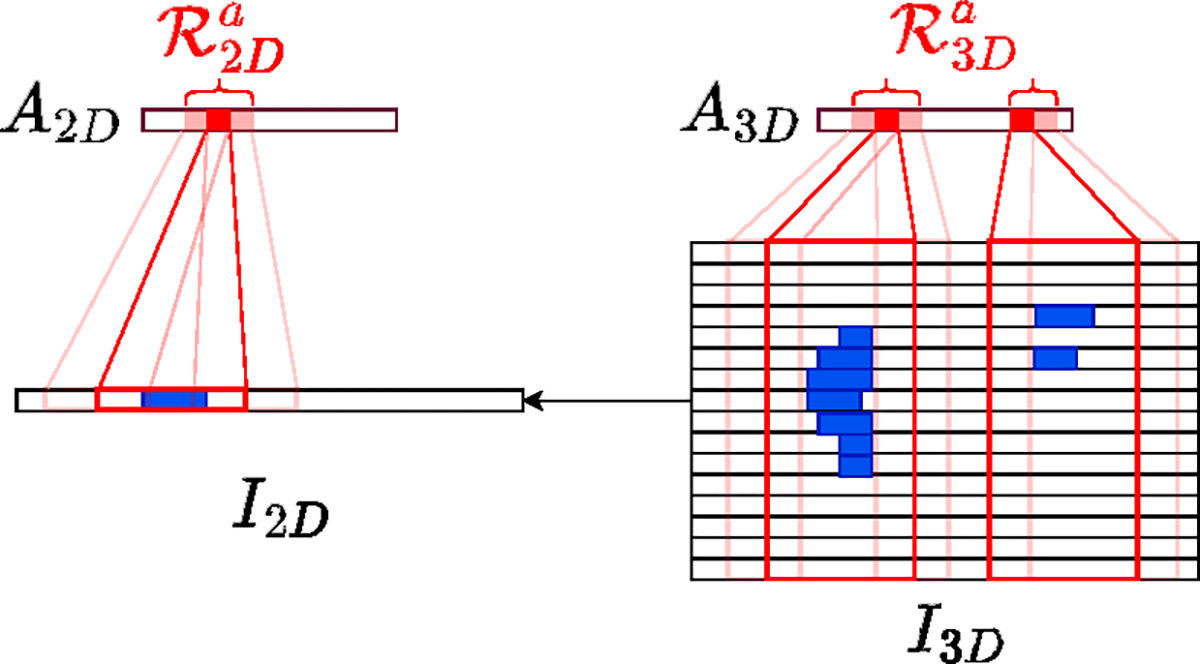
The figure demonstrates the relationship between the activated regions of the 2D and 3D branches, denoted by ${ℛ}_{2D}^{a}$ and ${ℛ}_{3D}^{a}$, respectively. The blue strips in the input images represent discriminative features such as edema or hemorrhage. The red boxes in $I_{2D}$ and $I_{3D}$ correspond to the receptive fields that activate the regions ${ℛ}_{2D}^{a}$ and ${ℛ}_{3D}^{a}$, respectively.

Since the 2D branch’s input $I_{2D}$ is a subset of the 3D branch’s input $I_{3D}$, it is expected that the 2D branch’s activated region ${ℛ}_{2D}^{a}$ in $A_{2D}$ is contained within the 3D branch’s activated region ${ℛ}_{3D}^{a}$. Similarly, the 3D branch’s non-activated region ${ℛ}_{3D}^{\text{na}}$ in $A_{3D}$ is expected to be contained within the 2D branch’s non-activated region ${ℛ}_{2D}^{na}$. This relationship is illustrated in equations (7), (8) and figure 7.

(7) $${ℛ}_{2D}^{a}\subset {ℛ}_{3D}^{a},$$

(8) $${ℛ}_{3D}^{na}\subset {ℛ}_{2D}^{na}.$$

The aforementioned relation can be regarded as prior knowledge for the classification of CT brain stacks. Next, we will show how we incorporate this ‘expected prior’ into our proposal CBAR-Net.

#### 2.4.4. Collaborative brain attention regularization (CBAR)

We proposed a CBAR to our CBAR-Net. CBAR has two parts: regularization on 2D branch ${ℒ}_{2D\text{,cbar}}$ and on 3D branch ${ℒ}_{3D,\text{cbar}}$.

**Figure 8.**
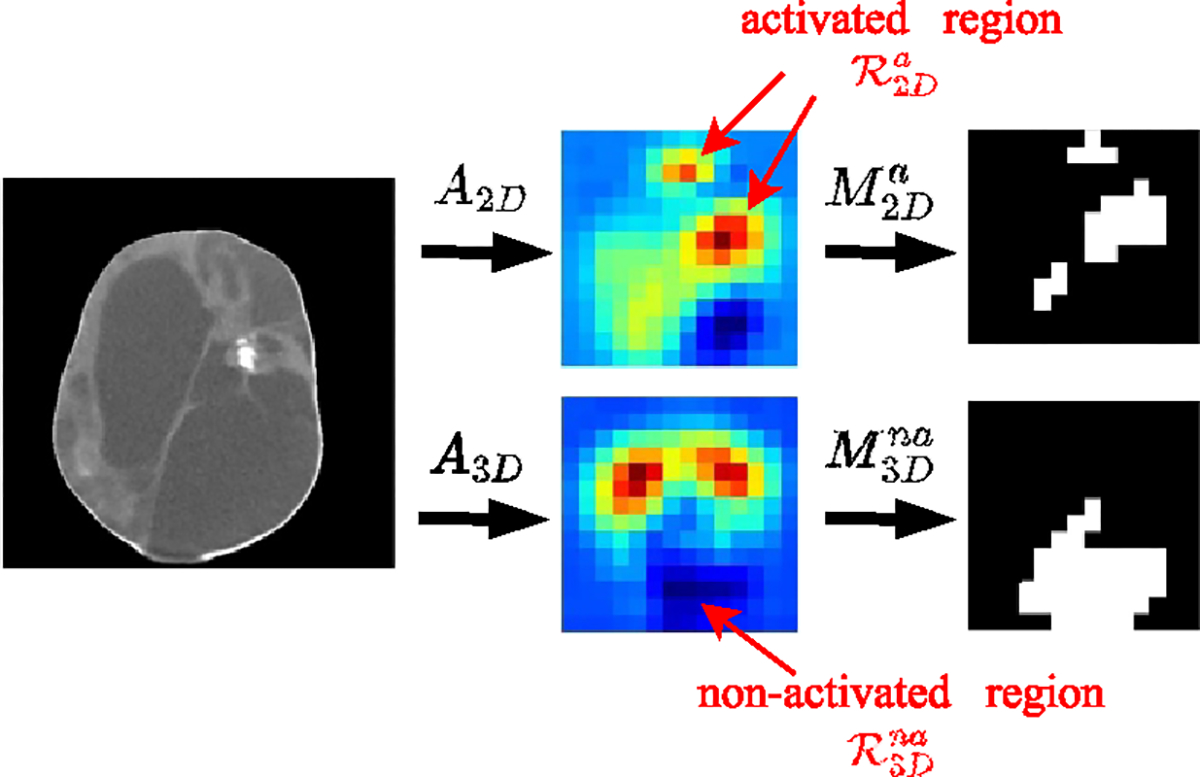
Generation of masks for activated and non-activated regions using CAMs from both the 2D and 3D branches.

First, the activated region ${ℛ}_{2D}^{a}$ and the non-activated region ${ℛ}_{3D}^{\text{na}}$ are selected via thresholding the CAMs. Next, the activated region mask of 2D branch and non-activated region mask of 3D branch are generated. The process is shown in figure 8.

For the 2D branch, its activation in the 3D branch’s non-activated region $R_{3D}^{\text{na}}$ will be suppressed via minimizing the following loss term:

(9) $${ℒ}_{2D,cbar}={∥M_{3D}^{\text{na}}⊙A_{2D}∥}_{1}.$$

For the 3D branch, its activation in the 2D branch’s activated region will be encouraged via minimizing the following loss term because of the minus sign:

(10) $${ℒ}_{3D,cbar}=-{∥M_{2D}^{a}⊙A_{3D}∥}_{1}.$$

In training, ${ℒ}_{2D,\text{cbar}}$ and ${ℒ}_{3D,\text{cbar}}$ appear as regularization terms along with the BCE loss term.

### 2.5. Loss function

The loss functions for 2D-branch, 3D-branch and fusion block are defined as:

(11) $${ℒ}_{\text{fusion}\;}={ℒ}_{BCE}$$

(12) $${ℒ}_{2D}={ℒ}_{BCE,2D}+\alpha_{1}{ℒ}_{2D,\text{cbar}}+\alpha_{2}{ℒ}_{\text{bar}}$$

(13) $${ℒ}_{3D}={ℒ}_{BCE,3D}+\alpha_{3}{ℒ}_{3D,cbar}.$$

The two branches of the CBAR-Net are designed to be trained alternately, meaning that either the 2D or 3D branch is fixed during a training period T.

The overall loss function for CBAR-Net ${ℒ}_{\text{cbar}-\text{n}}$ in iteration j within a training period T is defined as:

(14) $$\left\{\begin{array}{ll} {ℒ}_{\text{fusion}}+\beta {ℒ}_{2D},\;\text{if} & j\in \left\{0,1,\ldots ,\frac{T}{2}\right\}\\ {ℒ}_{\text{fusion}}+\beta {ℒ}_{3D},\;\text{if}\; & j\in \left\{\frac{T}{2},\frac{T}{2}+1,\ldots ,T\right\} \end{array}\right.$$

where n=0,1,2,… and T represents the number of iterations in a training period. The details of CBAR implementation along with code are explained in https://github.com/Schiff-Lab/Brain-CT-Image-Infection-Machine-Learning.

#### 2.5.1. Ensemble of predictions

**Figure 9.**
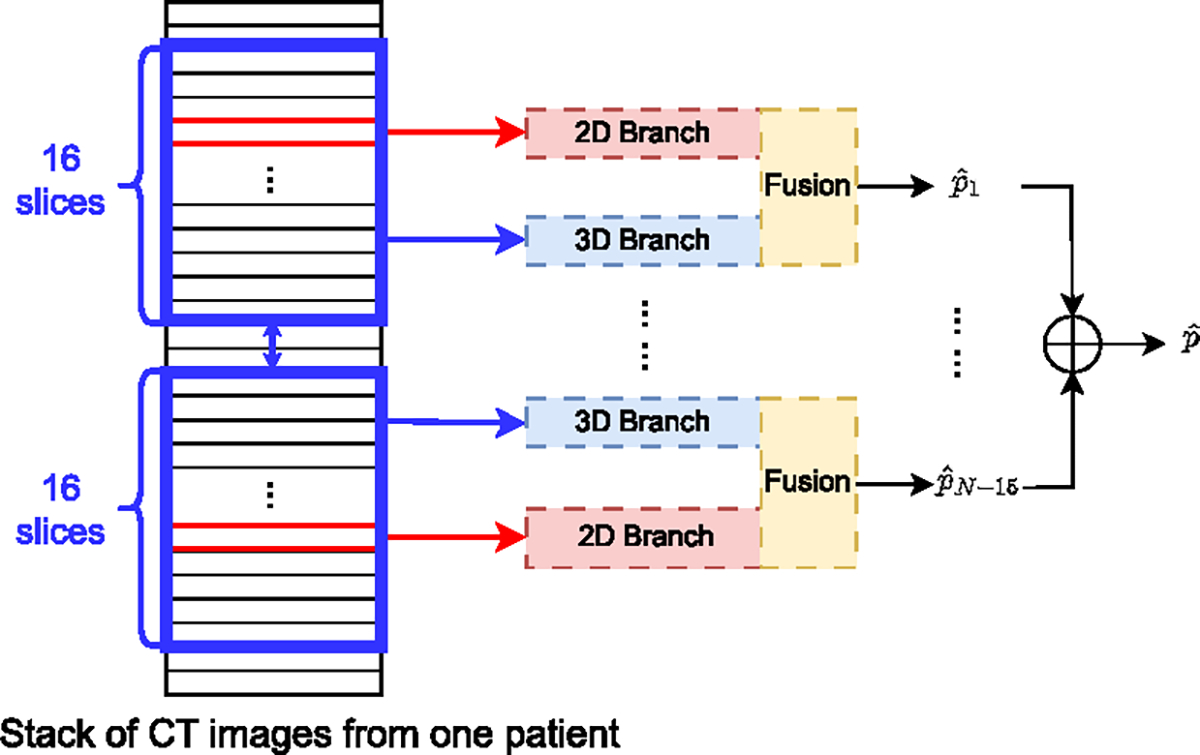
Inference via an ensemble mechanism. For each patient, multiple stack of images (16 slices in blue) are fed to 3D branch (blue box), and their middle slices are fed to 2D branches respectively. The outputs are fused later. Results from multiple stacks are averaged to form the final prediction.

Since each CT stack contains N slices, we can extract N-L inputs $I_{3D}^{i}(i=1,\ldots ,N-L)$, consisting of L slices, by sliding a window from the top to the base region of the stack. During the inference (testing) stage, the CBAR-Net outputs N-L probability scores ${\overset{ˆ}{p}}_{1},\ldots ,{\overset{ˆ}{p}}_{N-L}$, which are then averaged to obtain the final prediction, as shown in equation (15). The demonstration of the overall inference process is also illustrated in figure 9.

(15) $$\hat{p}=\frac{1}{N-L}\sum_{i=1}^{N-L}f_{\text{cbar}\;-\text{n}}\left(I_{3\text{D}}^{i}\right),$$

where $f_{\text{cbar}-n}(\cdot )$ is functional representation of CBAR-Net as shown in figure 6.

### 2.6. Experimental setting

#### 2.6.1. Training and evaluation configuration

For classification of PIH vs. NPIH, five-fold cross-validation was conducted independently 3 times on 387 patients. At each fold, 80% of the scans are assigned for training and 20% are assigned for evaluation. Similarly, for the classification of *Paenibacillus* vs. non-*Paenibacillus*, 206 scans with PIH are used with the 5-fold cross-validation scheme.

#### 2.6.2. Data augmentation

Several data augmentation operations are employed, including flipping, randomly rotating −20° to 20° in the axial plane, cropping and resizing, and adding Gaussian noise. One of these augmentations is randomly selected for each iteration of the training. The 2D slice is extracted from the augmented 3D sample maintaining the correspondence between the 2D and 3D samples.

#### 2.6.3. Implementation setting

We optimize the models using Adam [37] with a mini-batch size of 32 and 8 for the BAR-Net and CBAR-Net, respectively. We train each model for 100 epochs. The initial learning rate is set to be 0.0001 and is divided by a factor of 2 after every 30 epochs. The α in equation (5) is 0.0001. In equation (14), the β is 0.5 and T is 20. In equations (12) and (13), the $\alpha_{1}$ is 0.01, $\alpha_{2}$ is 0.0001 and $\alpha_{3}$ is 0.0001. We use weight decay of 0.000001 and a momentum of 0.9. The training and evaluation is carried out using Pytorch framework [38] on a NVIDIA TITAN X GPU.

## 3. Results

### 3.1. Evaluation metrics

We use accuracy, sensitivity and specificity to evaluate the performance of the models, which are defined as:

(16) $$\text{accuracy}=\frac{TP\;+\;TN}{TP\;+\;TN\;+\;FP\;+\;FN}$$

(17) $$\text{sensitivity}=\frac{TP}{TP\;+\;FN}$$

(18) $$\text{specificity}=\frac{TN}{TN\;+\;FP}$$

where TP, TN, FP, FN represent the number of true positive, true negative, false positive, and false negative findings. Additionally, the receiver operating characteristic (ROC) and area under the curve (AUC) are reported.

First, we analyze the effectiveness of the components in the BAR-Net and CBAR-Net. Second, we evaluate BAR-Net with CBAR-Net on classification of PIH/NPIH and *Paenibacillus/non-Paenibacillus* and compare the results against the SOTA methods. Finally, we show that our proposed BAR/CBAR-Net yield more interpretable activations than SOTA black-box deep networks.

### 3.2. Ablation study

Here we study the effectiveness of the components in both BAR/CBAR-Net. The results are reported in tables 1 and 2.

First, we study the impact of the BAR ${ℒ}_{\text{bar}}$ in BAR-Net under the training-validation configuration described in section 2.6.1 and in another low-training setup where 40% of the available training data is used. The experiment with low training setting is particularly important to examine the robustness of the model, i.e. the ability of adapting to new test samples and datasets beyond the training data. Table 1 shows that brain attention regularization $\left({ℒ}_{\text{bar}}\right)$ in BAR-Net offers accuracy improvements over using ${ℒ}_{BCE}$ alone that are particularly pronounced when the training set is smaller. A comprehensive evaluation of BAR/CBAR-Net against SOTA methods for varying training regimes is discussed in section 3.3.3.

In this study, we analyze the performance of using L=4,8,16,32 number of slices as the 3D branch’s input $I_{3D}$ in CBAR-Net. The structure of the 3D branch is slightly adjusted to ensure that the dimensions of $A_{3D}$ and $A_{2D}$ are the same, as described in section 2.4.2. Note that, according to equation (15), the size of L influences the number of outputs N-L used for making the final decision. As shown in table 2, L=16 achieves the best results compared to L=4,8,32. Using 4 or 8 slices does not show a significant gain over the 2D model (i.e. BAR-Net), while the result of using 32 slices is degraded compared to BAR-Net. The observations can be explained as follows: i) The most informative region is typically around the middle, which can be covered by most of the sub-volumes of 16 slices, as N is around 30 – 40 for most CT stacks. ii) Using too few slices, such as 4 or 8, as input might not capture enough inter-slice information. In contrast, using a larger number of slices, such as 32, increases the size of the input, resulting in higher dimensional training data without an increase in the number of training samples. This can lead to over-fitting.

### 3.3. Comparisons against SOTA methods

We compare against carefully selected SOTA methods, which are introduced here:

- Singh *et al* [8]: A method that uses traditional 3D networks for 3D medical image classification.
- Chen *et al* [27]: This method consists of 2 branches: a 2D CNN and a 3D CNN, which captures the features of multi-view 2D planes and the whole 3D brain. The features are then fused for final classification.
- Qiu *et al* [11]: This method trains a patch-wise 3D CNN outputting 3D probability maps which are mapped to the labels via a multi-layer perceptron.
- Gao *et al* [26]: A deep network for CT brain image classification is proposed that fuses 2D and 3D features from two networks.
- Yang *et al* [12]: This method reinvents 3D convolution via combination of 2D convolution.
- Li *et al* [9]: DenseNet [32] based method for classification of histopathology images but is broadly applicable.
- Jang and Hwang [10]: This method combines a CNN and a transformer for the task of 3D medical image classification.

#### 3.3.1. Evaluation of classification of PIH/NPIH

**Figure 10.**
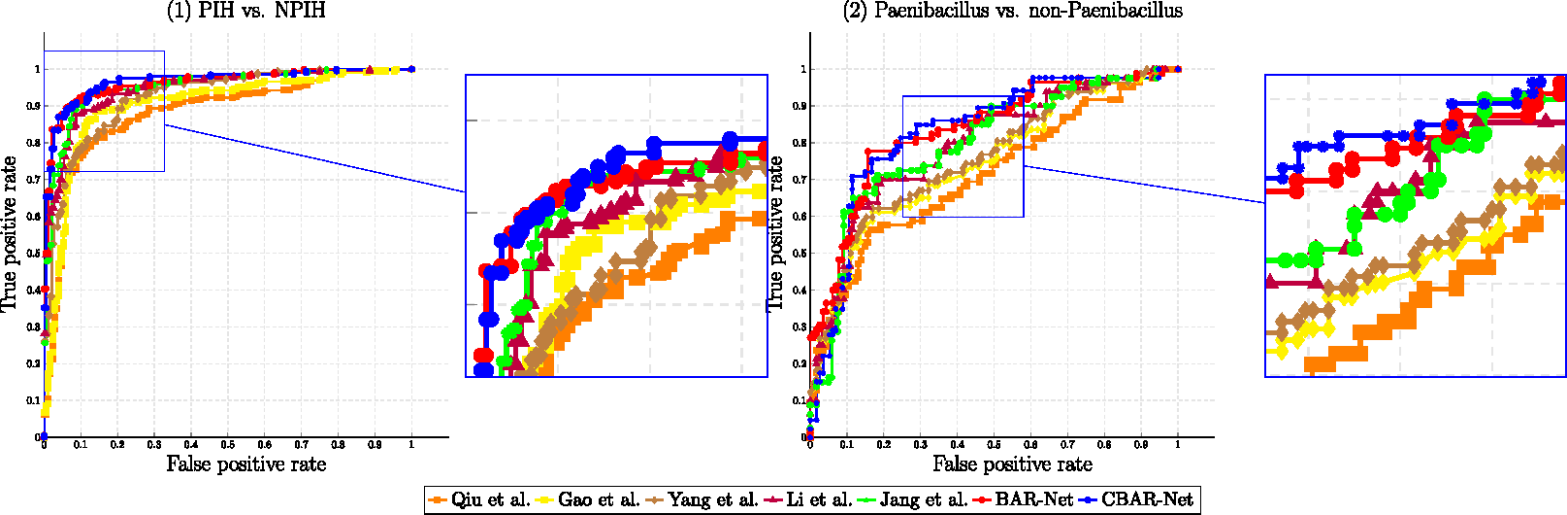
Comparisons of ROC curves output from BAR/CBAR-Net against SOTA methods.

We first evaluate the performance of our proposed methods on PIH verses NPIH classification against SOTA alternatives. The mean and standard deviation of accuracy, sensitivity, specificity results are shown in table 3. The ROC curve is illustrated in figure 10 and corresponding AUC is reported in the last column of table 3.

Overall, our proposed BAR-Net and CBAR-Net outperform SOTA methods on classification of PIH/NPIH in terms of accuracy, sensitivity, specificity, and AUC. First, the results showed that 3D methods may not necessarily lead to better performance primarily due to over-fitting and training stability issues. 2D methods with a powerful architecture and careful regularization can achieve competitive accuracy. Second, the results in table 3 confirm that a joint 2D/3D design with a composite loss function is an effective strategy.

#### 3.3.2. Evaluation of *Paenibacillus* detection

We conducted an evaluation of the performance of **(a) Evaluation of classifier 2: *Paenibacillus* detection within the PIH cohort:** table 4 presents the accuracy, sensitivity, and specificity of *Paenibacillus* detection by Classifier 2 within the PIH cohort. It is worth noting that the task of pathogen identification is considerably more challenging, as indicated by the relatively lower classification accuracy compared to table 3. This is to be expected because this task involves a more fine-grained classification within the PIH cohort. **(b) Evaluation of the over-all pipeline: *Paenibacillus* detection without access to ground truth PIH samples:** In real-world settings, the ground-truth for PIH/NPIH is unavailable and is predicted by Classifier 1. We report in table 5 the results of *Paenibacillus* detection for the entire dataset, representing the performance of the pipeline in figure 2. If a scan is classified as NPIH by Classifier 1 (i.e. the PIH/NPIH classifier), it is labeled as non-*Paenibacillus*. If a scan is classified as PIH, it is forwarded to Classifier 2 for *Paenibacillus* detection. For any misclassified NPIH scans that are identified as PIH by Classifier 1 in stage 1, we treat their ground truth label as non-*Paenibacillus* for evaluation. It is important to note that the test dataset, i.e. collection of scans (entire dataset) for the results in table 5 is therefore different from the test scans that correspond to the result in table 4—which only include scans within the PIH cohort using ground-truth knowledge. Hence, the results in tables 4 and 5 should *not be* directly compared. That said, the performance gap between (C)-BAR-Net and state-of-the-art (SOTA) methods in table 5 is wider, further emphasizing the merits of our domain enriched learning approach.

#### 3.3.3. Performance with low training setup

**Figure 11.**
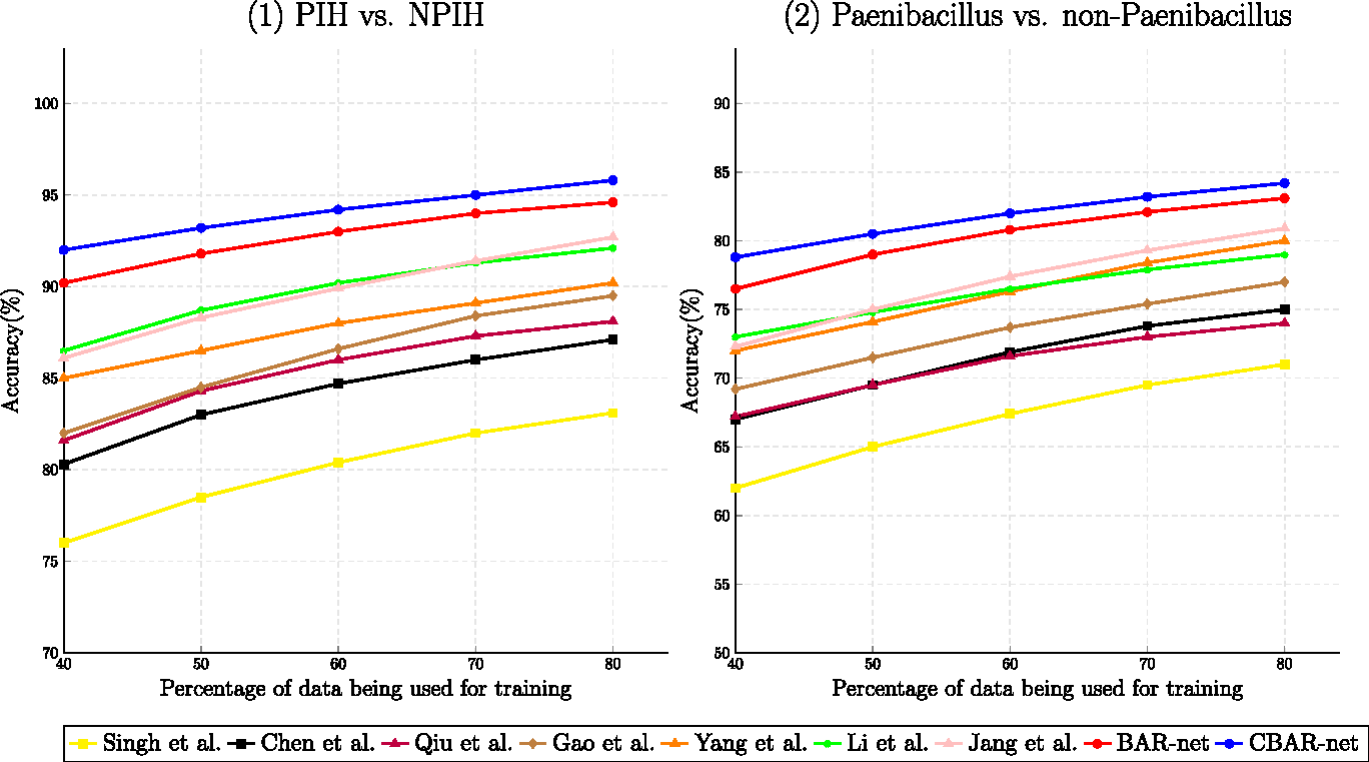
Illustration of BAR/CBAR-Nets’ and SOTA methods’ classification performance under variable training size. The proportion of training data in the whole dataset ranges from 40% to 80%. Note that BAR/CBAR-Net offers improved generalizability owing to our custom designed regularizers, as evidenced by a greater than 5% accuracy gain over the best of the SOTAs in the low-training regime.

A well-designed and robust machine learning should exhibit decent performance even in the face of limited training data. To examine training robustness, we report accuracy results for various percentages of available training samples. The training data utilized in this experiment varies from 40% to 80% of the original training size. The results are illustrated in figure 11. As can be observed, BAR-Net and CBAR-Net exhibit graceful degradation with respect to training size compared to other SOTA methods. This is because our proposed regularizers ${ℒ}_{\text{bar}}$ in equations (4) and (5) exploit expected output characteristics of CAMs, which are independent of the choice of training samples thereby improving robustness to the quantity and quality of available training images.

A statistical comparison with the best performing SOTA method [10] is shown in table 6. The statistical difference widens further for the low-training scenario between our proposed method and the existing SOTA method.

#### 3.3.4. Visualization of impact of regularization on CAMs

**Figure 12.**
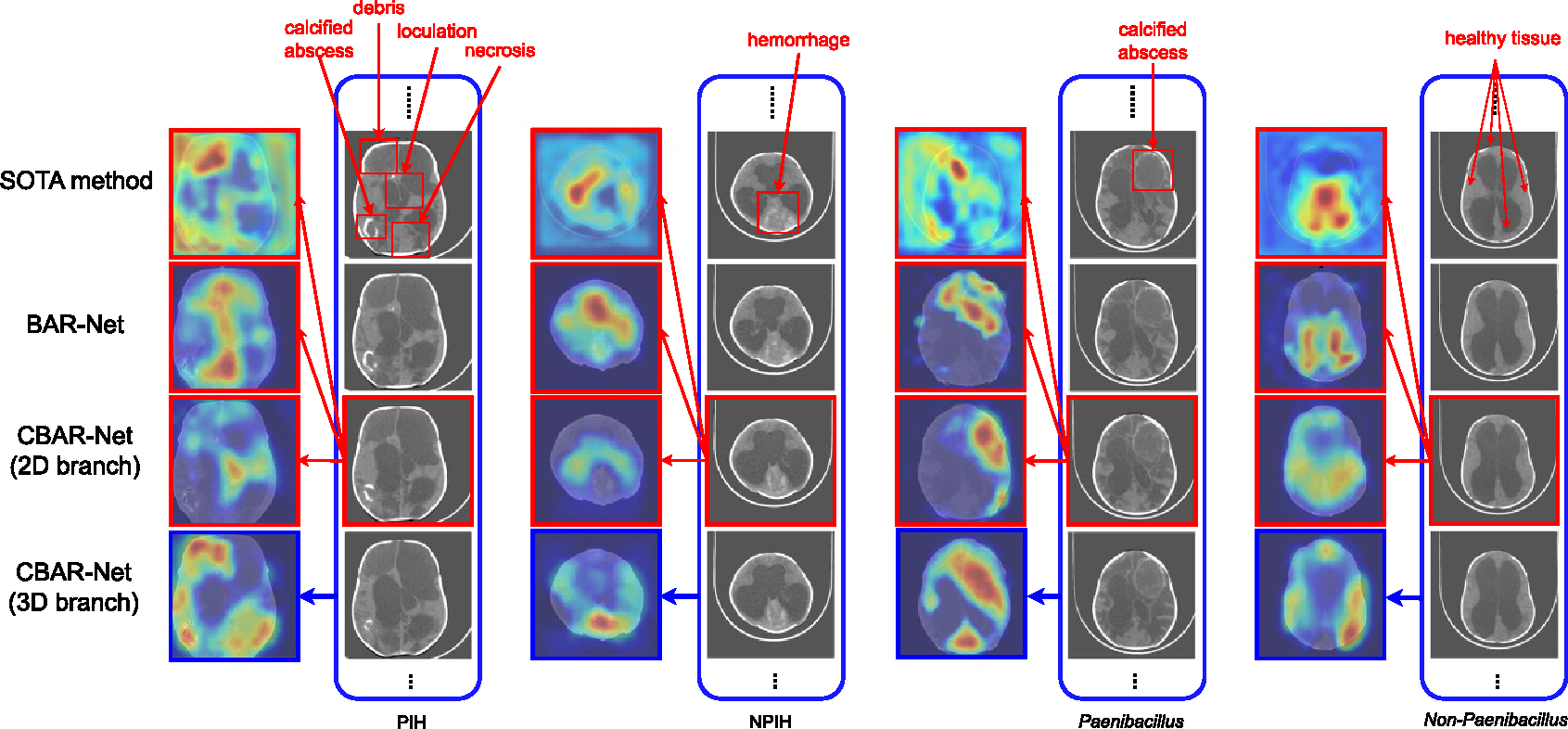
The CAMs (upscaled for visualization) of the best performing SOTA method [10], BAR-Net, 2D branch and 3D branch of CBAR-Net are shown. The CT image stack shown in each column are from examples of PIH, NPIH, *Paenibacillus*, and non-*Paenibacillus* cases from left to right respectively.

The impact of brain attention regularization on the output activation maps is demonstrated in figure 12. We compare CAMs from BAR-Net, CBAR-Net and the best of the SOTA methods in [10]. The results from the classification of PIH versus NPIH are shown in the left 2 groups while the right 2 groups are results from the classification of *Paenibacillus* versus non-*Paenibacillus*. For the CAMs, the first row shows the CAMs of the method in [10]. The second row shows the results of BAR-Net. The third and the last row are results from the 2D branch and the 3D branch of CBAR-Net respectively. The input of method [10], BAR-Net and the 2D branch of CBAR-Net are single slice (red frame), while the input of 3D branch of CBAR-Net is a stack of slices (blue frame). Note that even though the activation map from the 3D branch is a 2D map, its receptive field encompasses 3D structure which covers the local space along the *z*-axis. Thus, the activation shown in the 3D branch’s CAM is not only influenced by the present slice but also by the adjacent slices in its vicinity. For BAR and CBAR-Net, first we notice that all the activations are concentrated inside the brain region with the help of ${ℒ}_{\text{bar}}$ regularization in equation (4). Since no mask is provided as input in the inference phase, it indicates that the model learns to distinguish the brain from non-brain and suppress the regions outside the brain region. By doing so, the model will learn to focus on reliable discriminative regions. Secondly, we can observe that the CAMs from BAR-Net and CBAR-Net are significantly more interpretable than those from SOTA methods. BAR-Net is often able to highlight the pathological features that neuroscience experts may understand in their decision making process. In particular, CBAR-Net’s 3D branch is able to highlight infection regions which have high inter-slice correlation. With the help of CBAR-Net, calcifications and abcess are highlighted. The results indicate that the 3D branch is able to capture more information along the *z*-axis which the 2D branch is unable to acquire. Finally, when comparing activation maps of 2D and 3D branches, we observe that there are some overlapping regions indicating that the two branches reach agreement on certain features. However, there are also some non-overlapping activations, which indicates that the two branches capture diverse information.

## 4. Discussion and conclusion

This article presents a custom designed machine learning framework for the problem of infection diagnosis from hydrocephalus CT images. Differentiating infants with PIH and NPIH and determining the presence of pathogens such as *Paenibacillus* is crucial for subsequent treatment. However, typically such inference is made after microbial culture, or molecular diagnostic techniques, which can result in considerable delay to diagnosis and often fail for organisms that cannot be recovered in culture readily or were not included in testing panels [3, 40]. We present an automatic identification system based on CT images, which classifies PIH versus NPIH and detects *Paenibacillus* using deep learning AI models trained from data. The system is able to make decisions automatically and in real-time provide with patients’ brain images at point-of-care. The deep learning models have evident strengths in modeling complex non-linear mapping from input images to labels. That said, one of the major concerns, especially for clinicians, is interpretability of the algorithm’s decision making process. Hence, in this paper we studied CAMs [5] and developed a BAR mentioned in section 2.3. The proposed regularization term encourages the CNN model to pay attention to informative regions inside the brain, leading to more interpretable CAMs, which provides valuable information to clinicians. In addition to the 2D image classifier, a hybrid of 2D/3D models is developed to better capture the inter-slice correlation. To the best of our knowledge, our findings are unique among early efforts in interpretable AI-based models for classification of hydrocephalus and underlying pathogens using CT scans. The performance of the proposed BAR-Net and CBAR-Net is validated on real images against several SOTA methods. The results showed our developed system achieves 95.8% accuracy on the task of PIH/NPIH classification and 84% on *Paenibacillus*/non-*Paenibacillus* classification. Both outperform SOTA methods by a significant margin. In addition, CAMs shown in section 3.3.4 indicate their potential as useful reference for the diagnostic process. Currently, the classification of PIH/NPIH and *Paenibacillus*/non-*Paenibacillus* are conducted separately by two networks. In the future, combining two models into one via multi-task learning or transfer learning is a viable direction. Finally, while our *domain enriched* approach produces more reliable activation maps compared to other SOTA methods, obtaining ‘optimal’ and fully interpretable activations remains an open challenge. A particularly interesting direction is where activations predicted by a deep network may be treated as feedback to the expert clinicians, such that collaborative interactions between the algorithm and human expert can be facilitated. Achieving the above may involve newer learning frameworks and forms an exciting future research frontier.

## Figures and Tables

**Table 1.** Brain attention regularization’s impact on BAR-Net’s performance on classification of PIH/NPIH.

Loss function	Size of training set	Accuracy

${ℒ}_{BCE}$	100%	93.5 %
${ℒ}_{BCE}\;+\;\alpha {ℒ}_{bar}$	100%	94.3 %
${ℒ}_{BCE}$	40%	86.7 %
${ℒ}_{BCE}\;+\;\alpha {ℒ}_{bar}$	40%	90.4 %

**Table 2.** Ablation study: impact of number of slices in CBAR-Net’s input on PIH/NPIH classification.

CBAR-Net					
	
	3D branch’s input: *L* × *H* × *W*				
		
2D branch	*L* = 4	*L* = 8	*L* = 16	*L* = 32	Accuracy

✓					94.3%
✓	✓				94.1%
✓		✓			94.7%
✓			✓		95.8%
✓				✓	93.2%

**Table 3.** Classification result of PIH verses NPIH on CURE dataset.

Methods	Accuracy(%)	Sensitivity(%)	Specificity(%)	AUC

Singh *et al* [8] (3D)	83.3 ± 3.11	82.7 ± 3.15	85.2 ± 3.73	0.844 ± 0.020
Chen *et al* [27] (hybrid)	87.6 ± 2.39	86.1 ± 2.86	89.2 ± 2.83	0.902 ± 0.016
Qiu *et al* [11] (3D)	88.1 ± 2.49	89.1 ± 3.16	87.3 ± 2.52	0.892 ± 0.016
Gao *et al* [26] (hybrid)	89.5 ± 2.41	87.2 ± 2.35	90.2 ± 2.63	0.903 ± 0.016
Yang *et al* [12] (multi-plane)	90.2 ± 2.19	91.3 ± 2.26	90.4 ± 2.34	0.921 ± 0.018
Li *et al* [9] (2D)	92.1 ± 1.81	91.5 ± 1.96	92.6 ± 2.28	0.941 ± 0.017
Jang *et al* [10] (multi-plane)	92.7 ± 2.07	91.8 ± 2.83	93.6 ± 2.96	0.937 ± 0.016

BAR-Net	94.3 ± 1.38	93.7 ± 1.81	95.4 ± 2.34	0.962 ± 0.012
CBAR-Net	95.8 ± 1.71	95.1 ± 2.13	96.4 ± 2.37	0.975 ± 0.011

**Table 4.** Classification result of *Paenibacillus* verses non-*Paenibacillus* on CURE dataset.

Methods	Accuracy(%)	Sensitivity(%)	Specificity(%)	AUC

Singh *et al* [8] (3D)	71.3 ± 4.11	69.7 ± 4.55	74.2 ± 5.56	0.732 ± 0.020
Chen *et al* [27] (hybrid)	75.8 ± 4.39	72.1 ± 3.96	78.2 ± 4.89	0.771 ± 0.021
Qiu *et al* [11] (3D)	74.2 ± 3.81	71.4 ± 5.14	77.6 ± 6.87	0.762 ± 0.019
Gao *et al* [26] (hybrid)	77.6 ± 3.10	72.3 ± 6.32	83 ± 5.65	0.782 ± 0.021
Yang *et al* [12] (multi-plane)	80.5 ± 3.19	76.3 ± 3.76	84.4 ± 3.88	0.815 ± 0.018
Li *et al* [9] (2D)	79 ± 3.18	73 ± 4.45	85.1 ± 3.39	0.812 ± 0.016
Jang *et al* [10] (multi-plane)	80.9 ± 2.94	74.1 ± 3.12	86.6 ± 2.98	0.821 ± 0.015

BAR-Net	82.8 ± 2.43	78.6 ± 3.32	87.9 ± 3.26	0.835 ± 0.015
CBAR-Net	84 ± 3.21	79.7 ± 3.49	89.1 ± 3.22	0.853 ± 0.016

**Table 5.** Classification result of *Paenibacillus* verses non-*Paenibacillus* on CURE dataset given the results of PIH/NPIH classification.

Methods	Accuracy (%)	Sensitivity (%)	Specificity (%)

Jang *et al* [10]	84.3 ± 3.24	72.1 ± 3.12	91.3 ± 3.17
BAR-Net	87.3 ± 3.20	77.6 ± 3.42	93.2 ± 3.34
CBAR-Net	88.1 ± 3.41	76.7 ± 3.79	93.8 ± 3.42

**Table 6.** T-test results following normality testing [39] for PIH and pathogen classification for both high and low training size.

	PIH classification				Pathogen classification			
		
	80% of data		40% of data		80% of data		40% of data	
				
Methods	*t*-value	*p*-value	*t*-value	*p*-value	*t*-value	*p*-value	*t*-value	*p*-value

BAR-Net and SOTA method [10]	2.902	0.007 152	5.228	1.49 × 10^−5^	2.032	0.0364	2.56	0.016
CBAR-Net and SOTA method [10]	4.34	0.000 167	6.244	9.52 × 10^−7^	2.87	0.0078	3.1448	0.003 913

## Data Availability

The data cannot be made publicly available upon publication because they contain sensitive personal information. The data that support the findings of this study are available upon reasonable request from the authors.
